# Description of the COVID-19 epidemiology in Malaysia

**DOI:** 10.3389/fpubh.2024.1289622

**Published:** 2024-03-13

**Authors:** Mohamad Nadzmi Md Nadzri, Ahmed Syahmi Syafiq Md Zamri, Sarbhan Singh, Mohd Ghazali Sumarni, Chee Herng Lai, Cia Vei Tan, Tahir Aris, Hishamshah Mohd Ibrahim, Balvinder Singh Gill, Nur’Ain Mohd Ghazali, Nuur Hafizah Md Iderus, Mei Cheng Lim, Lonny Chen Rong Qi Ahmad, Mohd Kamarulariffin Kamarudin, Nur Ar Rabiah Ahmad, Kok Keng Tee, Asrul Anuar Zulkifli

**Affiliations:** ^1^Institute for Medical Research (IMR), National Institutes of Health (NIH), Ministry of Health Malaysia, Setia Alam, Malaysia; ^2^Ministry of Health Malaysia, Putrajaya, Malaysia; ^3^Department of Medical Microbiology, Faculty of Medicine, Universiti Malaya, Kuala Lumpur, Malaysia; ^4^International Medical School, Management and Science University, Shah Alam, Selangor, Malaysia

**Keywords:** COVID-19, epidemiology, outbreak, variant, Cases

## Abstract

**Introduction:**

Since the COVID-19 pandemic began, it has spread rapidly across the world and has resulted in recurrent outbreaks. This study aims to describe the COVID-19 epidemiology in terms of COVID-19 cases, deaths, ICU admissions, ventilator requirements, testing, incidence rate, death rate, case fatality rate (CFR) and test positivity rate for each outbreak from the beginning of the pandemic in 2020 till endemicity of COVID-19 in 2022 in Malaysia.

**Methods:**

Data was sourced from the GitHub repository and the Ministry of Health’s official COVID-19 website. The study period was from the beginning of the outbreak in Malaysia, which began during Epidemiological Week (Ep Wk) 4 in 2020, to the last Ep Wk 18 in 2022. Data were aggregated by Ep Wk and analyzed in terms of COVID-19 cases, deaths, ICU admissions, ventilator requirements, testing, incidence rate, death rate, case fatality rate (CFR) and test positivity rate by years (2020 and 2022) and for each outbreak of COVID-19.

**Results:**

A total of 4,456,736 cases, 35,579 deaths and 58,906,954 COVID-19 tests were reported for the period from 2020 to 2022. The COVID-19 incidence rate, death rate, CFR and test positivity rate were reported at 1.085 and 0.009 per 1,000 populations, 0.80 and 7.57%, respectively, for the period from 2020 to 2022. Higher cases, deaths, testing, incidence/death rate, CFR and test positivity rates were reported in 2021 and during the Delta outbreak. This is evident by the highest number of COVID-19 cases, ICU admissions, ventilatory requirements and deaths observed during the Delta outbreak.

**Conclusion:**

The Delta outbreak was the most severe compared to other outbreaks in Malaysia’s study period. In addition, this study provides evidence that outbreaks of COVID-19, which are caused by highly virulent and transmissible variants, tend to be more severe and devastating if these outbreaks are not controlled early on. Therefore, close monitoring of key epidemiological indicators, as reported in this study, is essential in the control and management of future COVID-19 outbreaks in Malaysia.

## Introduction

The emergence of the COVID-19 infection caused by the SARS-CoV-2 virus was first reported in Wuhan, China, in late December 2019 (1). Within a short period since its discovery, it spread rapidly across many countries globally. The World Health Organization (WHO) subsequently declared COVID-19 a Public Health Emergency of International Concern (PHEIC) on 30 January 2020 (2). The COVID-19 public health emergency lasted for over 3 years till WHO declared it over on 5 May 2023. During this time, COVID-19 had infected almost 766 million individuals, which resulted in 6.9 million deaths globally (3).

In Malaysia, the first case of COVID-19 was reported on 25 January 2020, which marked the beginning of the pandemic, which lasted from 25 January 2020 till the disease was declared endemic in Malaysia on 1 April 2022. There was a total of 4 outbreaks caused by various COVID-19 variants over the 2-year period. These outbreaks were caused by the following 4 COVID-19 variants: Wuhan, Beta, Delta, and Omicron. Subsequently, each outbreak was then named corresponding to the circulating variants. The first outbreak by the Wuhan variants lasted for 29 weeks, from January 2020 to August 2020 [Epidemiological Week (Ep Wk)9/2020 to 37/2020] (4). This outbreak was characterized by a high case fatality rate and was when the Public Health Social Measure (PHSM), which included movement restrictions, was initiated.

The second Beta variant outbreak occurred from September 2020 to March 2021 (Ep Wk 37/2020 to 13/2021), which lasted for a duration of 30 weeks (5). This was followed by the highly virulent Delta outbreak from April 2021 to January 2022, which lasted for 43 weeks from (Ep Wk 13/2021 to 3/2022) (6). The Delta outbreak was predominantly a severe outbreak with high case burden and mortality. In addition, it was during this outbreak that the introduction of the COVID-19 vaccine commenced. The fourth outbreak, which was caused by the Omicron variant, marked the point this disease was declared endemic in Malaysia (7). The Omicron outbreak occurred from January 2022 till the disease was declared endemic in April 2022 (Ep Wk 3/2022 to 18/2022).

As the disease evolved, the demographic characteristics changed due to the various evolving variants requiring specific interventions and control measures. Hence, it is essential to examine each outbreak’s demographic, epidemiological characteristics, and trends to understand the pandemic, which would subsequently assist in improving the management and control of the disease. In addition, a detailed description of each outbreak variant during the pandemic would enable us to understand better the progression, evolution, and severity of the various COVID-19 outbreak variants (8–10). Furthermore, by systematically analyzing these epidemiological indicators, evidence of the effectiveness of pharmaceutical and non-pharmaceutical outbreak-based control measures would assist the surveillance system in monitoring the pandemic. Hence, the knowledge of the disease evolution is important in the process of initiating and adjusting PHSM and vaccination (11–13).

To date, limited published studies have described epidemiological indicators and their trends during each COVID-19 outbreak during the pandemic in Malaysia (14). Therefore, the main aim of this study is to describe and compare the epidemiological indicators during the Wuhan, Beta, Delta, and Omicron outbreak from 2020 (Ep Wk 4/2020 to 18/2022) in terms of their disease demographics (case incidences and mortality rate), hospital admissions [intensive care unit (ICU) admissions, ventilated cases] and diagnostic testing (testing capacity, test positivity rates). This paper will provide a comprehensive analysis and understanding of the various epidemiological indicators and their progression during the COVID-19 pandemic, which would provide a more comprehensive knowledge of the disease evolution and comparison of distinct characteristics during each outbreak in Malaysia (15).

## Materials and methods

### Data source

Data on COVID-19 cases, deaths, ICU admissions, ventilator requirements and tests was sourced from the GitHub repository (MoH-Malaysia/covid19-public) and the Ministry of Health official COVID-19 website^1^^,^^2^ (16, 17). The study period was from the beginning of the COVID-19 outbreak in Malaysia, which began during Ep Wk 4/2020, to when it was declared endemic, corresponding to the end of the Omicron outbreak in Ep Wk 18/2022 (18). Daily COVID-19 data were aggregated based on epidemiological week (which starts on Sunday) for each outbreak (i.e., Wuhan, Beta, Delta and Omicron) at the national level (19).

### Data analysis

Data was analyzed using the Statistical Package for the Social Sciences (SPSS) version 26.0 (20). Table 1 shows percentages and frequencies of cases, deaths, average ICU admissions, average ventilator requirements, laboratory-confirmed test incidence rate, death rate, CFR, and test positivity rate. Average weekly incidence and death rate was estimated to allow for comparison between outbreaks. In addition, the age-gender-specific case, death, incidence rate, death rate and CFR were estimated. A 10-year interval was used to represent the age distribution as follows: 0–9 years, 10–19 years, 20–29 years, 30–39 years, 40–49 years, 50–59 years, 60–69 years, 70–79 years and more than 80 years. The analysis was done for the overall duration of the COVID-19 outbreak in Malaysia from Ep Wk 4/2020 to Ep Wk 18/2022, and for each outbreak of COVID-19, namely the Wuhan (Ep Wk 9/2020 to 37/2020), Beta (Ep Wk 37/2020 to 13/2021), Delta (Ep Wk 13/2021 to 3/2022), and Omicron (Ep Wk 3/2022 to 18/2022), and presented in tabular and time series plots.

**Table 1:** Epidemiological indicators.

No.	Epidemiological indicator	Formula
1	Incidence rate*	$\frac{\mathrm{Number}\;\mathrm{of}\;\mathrm{COVID}-19\;\mathrm{cases}}{\mathrm{Total}\;\mathrm{Population}}$ × 100,000
2	Case Fatality rate (CFR)	$\frac{\mathrm{Number}\;\mathrm{of}\;\mathrm{COVID}-19\;\mathrm{deaths}\;}{\mathrm{Total}\;\mathrm{COVID}-19\;\mathrm{cases}}$ × 100
3	Test positivity rate	$\frac{\mathrm{Number}\;\mathrm{of}\;\mathrm{positive}\;\mathrm{case}\;}{\mathrm{Total}\;\mathrm{tests}\;\left(RT-PCR\;\mathrm{and}\;RTK-Ag\right)}$ × 100
4	Death rate*	$\frac{\mathrm{Number}\;\mathrm{of}\;\mathrm{COVID}-19\;\mathrm{deaths}\;}{\mathrm{Total}\;\mathrm{Population}}$ × 100,000

*Rates were calculated based on weekly averages; Gender-Age specific incidence and death rates were calculated per 1,000 population.

## Results

### Cases and incidence rate

#### Overall

There was a total of 4,456,736 cases reported for the overall study period in which the highest and lowest weekly COVID-19 cases were reported in Ep Wk 10/2022 (*n* = 205,864) and Ep Wk 8/2020 (*n* = 0), respectively. The overall weekly average COVID-19 incidence rate was 108.51 per 100,000 population, as shown in Table 2.

**Table 2:** COVID-19 cases, deaths, incidence rate, death rate, CFR, testing capacity and test positivity rate by COVID-19 variants outbreaks in Malaysia.

	COVID-19 Waves COVID				Total (Ep Wk 4/2020 to Ep Wk 18/2022)
	Wuhan (Ep Wk 9/2020 to Ep Wk 37/2020)	Beta (Ep Wk 37/2020 to Ep Wk 13/2021)	Delta (Ep Wk 13/2021 to Ep Wk 3/2022)	Omicron (Ep Wk 3/2020 to Ep Wk 18/2022)	
Cases, N (%)	9,375 (0.19)	333,488 (7.48)	2,467,804 (55.37)	1,646,047 (36.93)	4,456,736
Deaths, N (%)	128 (0.34)	1,132 (3.18)	30,549 (85.86)	3,770 (10.60)	35,579
Average weekly incidence rate*	1.48	32.91	169.89	304.53	108.51
Average weekly death rate*	0.02	0.11	2.10	0.70	0.87
CFR (%)	1.42	0.34	1.24	0.23	0.80
Total test, N (%)	853,379 (1.45)	8,781,144 (14.91)	33,603,291 (57.04)	15,157,523 (25.73)	58,906,954
Test positivity rate (%)	1.00	3.80	7.34	10.86	7.57

*Weekly incidences /death rates were estimated and averaged over the number of Epidemiological Weeks for each outbreak.

#### By outbreaks

During the Wuhan outbreak, a total of 9,375 (0.19%) cases were reported, with the highest and lowest weekly COVID-19 cases being reported in Ep Wk 14/2020 (*n* = 1,163) and Ep Wk 8/2020 (*n* = 0), respectively. On the other hand, during the Beta outbreak, a total of 333,488 (7.48%) cases were reported, with the highest and lowest weekly COVID-19 cases being reported in Ep Wk 4/2021 (*n* = 29,206) and Ep Wk 38/2020 (*n* = 299) respectively. For the Delta outbreak, a total of 2,467,804 (55.37%) cases were reported, with the highest and lowest weekly COVID-19 cases being reported in Ep Wk 33/2021 (*n* = 150,933) and Ep Wk 13/2021 (*n* = 8,968) respectively.

As for the Omicron outbreak, a total of 1,646,047 (36.93%) cases were reported, with the highest and lowest weekly COVID-19 cases being reported on Ep Wk 10/2022 (*n* = 205,864) and Ep Wk 18/2022 (*n* = 8,732) respectively. Overall, the highest number of cumulative cases was reported during the Delta (*n* = 2,467,804, 55.37%) outbreak, followed by the Omicron (*n* = 1,646,047, 36.93%), Beta (333,488, 7.48%) and Wuhan (8,493, 0.19%) outbreak, respectively. The average weekly incidence rate for the Wuhan, Beta, Delta and Omicron outbreaks was 1.48, 32.91,169.89 and 304.53 per 100,000 populations, respectively.

#### Trends

**Figure 1:**
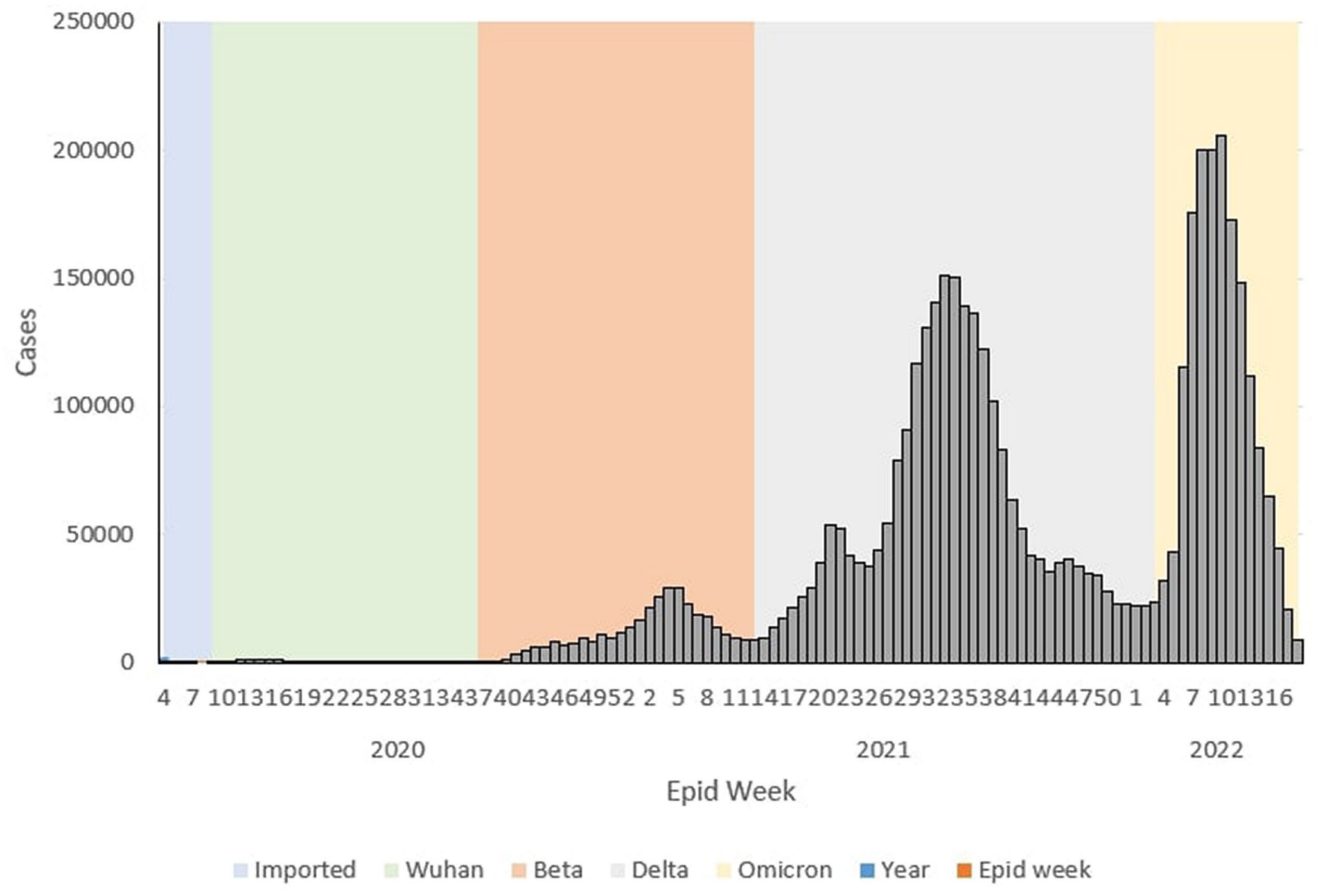
Weekly COVID-19 cases by outbreaks in Malaysia, 2020 to 2022*.

During the Wuhan outbreak, the epidemiological curve represented a slow increase in which the number of cases started to increase from Ep Wk 10/2020 (*n* = 68) and peaked in Ep Wk 14/2020 (*n* = 1,163). Following this, during the Beta outbreak, the epidemiological curve peaked on Ep Wk 4/2021 (*n* = 29,206). This was followed by the Delta outbreak, where the epidemiological curve peaked at Ep Wk 33/2021 (*n* = 150,933). Subsequently, during the Omicron outbreak, the epidemiological curve rose rapidly and peaked at Ep Wk 10/2022 (*n* = 205,864), corresponding to the highest number of weekly cases reported through the study duration in Malaysia (Figure 1).

### ICU admission and ventilator requirements

#### ICU admission trends

**Figure 2:**
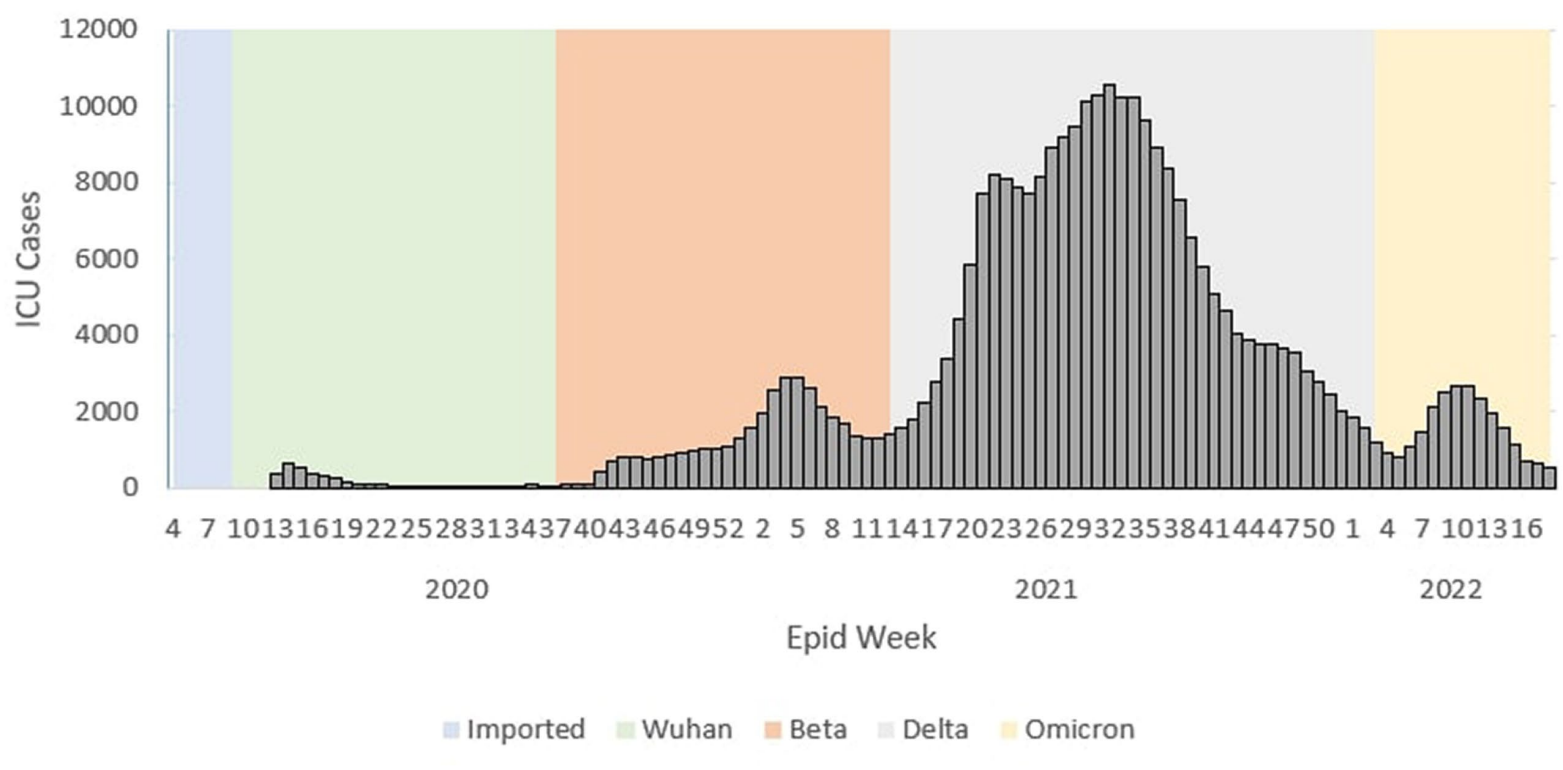
Weekly COVID-19 average ICU cases by outbreaks in Malaysia, 2020 to 2022.

During the Wuhan outbreak, the average number of cases requiring ICU admission started to increase from Ep Wk 13/2020 (*n* = 369) and peaked in Ep Wk 15/2020 (*n* = 549). Subsequently, the average ICU case trends decreased from Ep Wk16/2020 (*n* = 374) to Ep Wk36/2020 (*n* = 35). Following this, the Beta outbreak began where the average ICU case trends started to increase from Ep Wk37/2020 (*n* = 53) to Ep Wk 4/2021 (*n* = 2,902), corresponding to the longest increasing average ICU case trends of 21 weeks. A downward trend of the average ICU cases was observed from Ep Wk 5/2021 (*n* = 2,873) to Ep Wk 15/2021 (*n* = 1,775). This was followed by the Delta outbreak, where in the ICU case trend started to increase at Ep Wk 16/2021 (*n* = 2,024) to Ep Wk 32/2021 (*n* = 10,586) and subsequently decreased from Ep Wk 35/2021 (*n* = 9,637) until Ep Wk 5/2022 (*n* = 810) which corresponded to the longest decreasing average ICU case trends of 23 weeks. Following this, the Omicron outbreak began where the average ICU case trend started to increase at Ep Wk 6/2022 (*n* = 1,047) to Ep Wk 15/2022 (*n* = 1,102) and subsequently decreased at Ep Wk 16/2022 (*n* = 702) till Ep Wk 18/2022 (*n* = 506) as shown in Figure 2.

#### Ventilation requirement trend

During the Wuhan outbreak, the average number of ICU cases requiring ventilation increased from Ep Wk 13/2020 (*n* = 252) and peaked in Ep Wk 14/2020 (*n* = 381). Subsequently, the average ventilated case trends decreased from Ep Wk 15/2020 (*n* = 279) to Ep Wk 33/2020 19 (*n* = 4). Following this, the Beta outbreak began where the average ventilated case trends started to once again increase from Ep Wk 34/2020 (*n* = 22) until Ep Wk 5/2021 (*n* = 1,482), and this corresponded to the longest increasing average ventilated case trends of 25 weeks. A downward trend of average ventilated cases was observed from Ep Wk 6/2021 (*n* = 1,270) until Ep Wk 13/2021 (*n* = 750).

**Figure 3:**
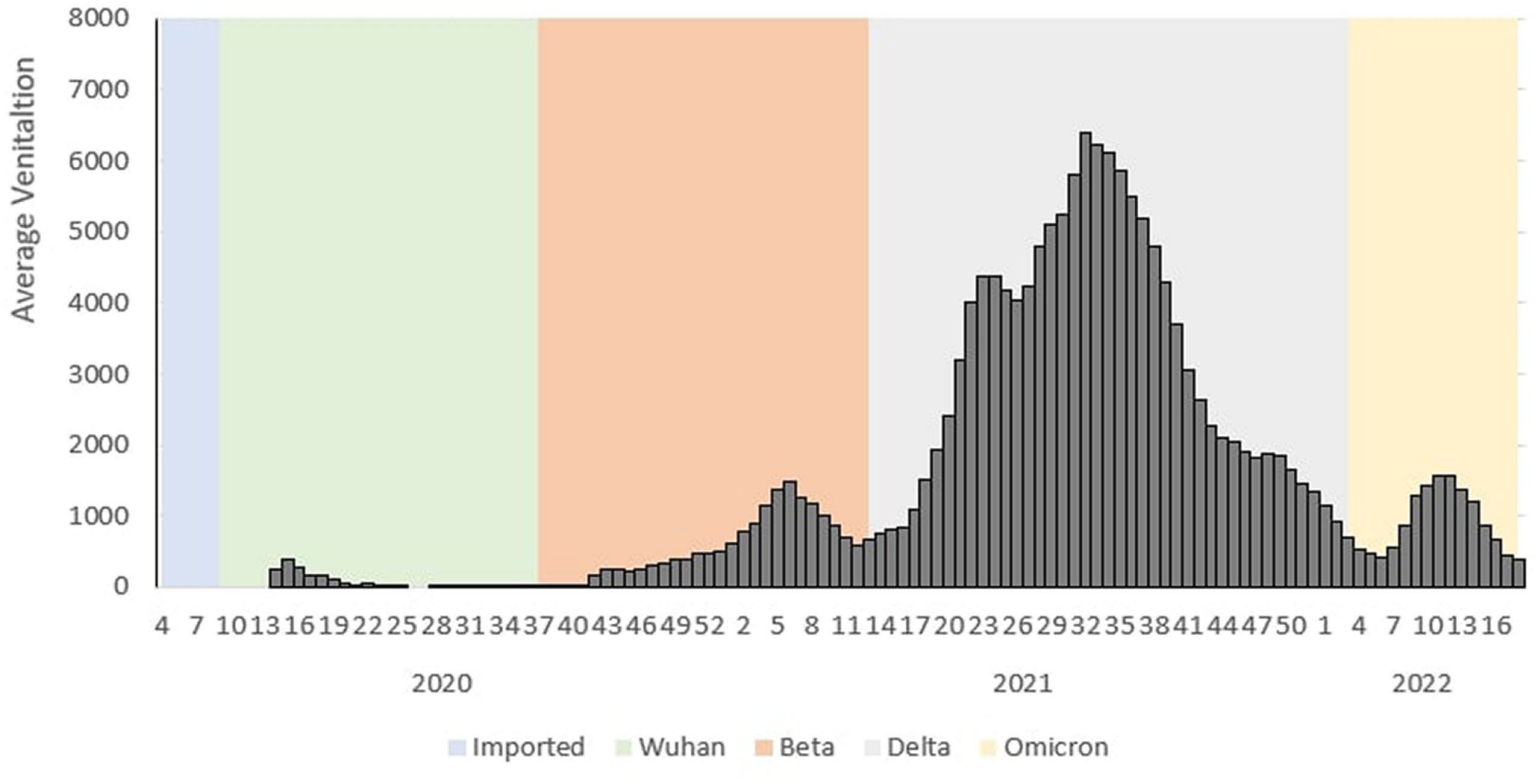
Weekly COVID-19 average ventilated cases by outbreaks in Malaysia, 2020 to 2022.

This was followed by the Delta outbreak, wherein the average number of ICU cases requiring ventilation increased from Ep Wk 14/2021 (*n* = 811) and peaked in Ep Wk 31/2021 (*n* = 6,388). The average ventilated case trends decreased from Ep Wk 32/2021 (*n* = 6,217) until Ep Wk 5/2022 (*n* = 420), corresponding to the longest decreasing average ventilated case trend of 26 weeks. Following this, the Omicron outbreak began when the average ventilated cases started to increase Ep Wk 6/2022 (*n* = 558) till Ep Wk 11/2022 (*n* = 1,558) and subsequently decreased from Ep Wk 12/2022 (*n* = 1,377) till Ep Wk 18/2022 (*n* = 297) as shown in Figure 3.

### Deaths, death rate, and CFR

#### Overall

There was a total of 35,579 deaths reported for the overall study period, in which the highest weekly COVID-19 death was reported in Ep Wk 37/2021 (*n* = 2,647), and no deaths were reported for several epidemiological weeks (17 weeks), respectively. The overall weekly average COVID-19 death rate and CFR were 0.87 per 100,000 population and 0.80%, respectively, as shown in Table 2.

#### By outbreak

During the Wuhan outbreak, a total of 128 (0.34%) deaths were reported, with the highest and lowest weekly COVID-19 deaths being reported in Ep Wk 13/2020 (*n* = 27) and several Epidemiological Weeks (6 weeks) with no deaths, respectively. While during the Beta outbreak, a total of 1,132 (3.18%) deaths were reported, with the highest and lowest weekly COVID-19 deaths being reported in week Ep Wk 5/2021 (*n* = 111) and Ep Wk 38 and 39/2020 (*n* = 3), respectively. For the Delta outbreak, a total of 30,549 (85.86%) deaths were reported, with the highest and lowest weekly COVID-19 deaths being reported in Ep Wk 37/2021 (*n* = 2,647) and Ep Wk 14/2021 (*n* = 35), respectively.

As for the Omicron outbreak, a total of 3,770 (10.6%) deaths were reported, with the highest and lowest weekly COVID-19 deaths being reported in Ep Wk 11/2022 (*n* = 609) and Ep Wk 18/2022 (*n* = 32), respectively. Overall, the highest number of cumulative deaths was reported during the Delta outbreak (*n* = 30,549, 85.86%), followed by the Omicron (*n* = 3,770, 10.60%), Beta (*n* = 1,132, 3.18%) and Wuhan (*n* = 128, 0.34%) outbreak, respectively. The CFR for the Wuhan, Beta, Delta and Omicron outbreaks was 1.42, 0.34, 1.24, and 0.23%, respectively, as shown in Table 2.

#### Trends

**Figure 4:**
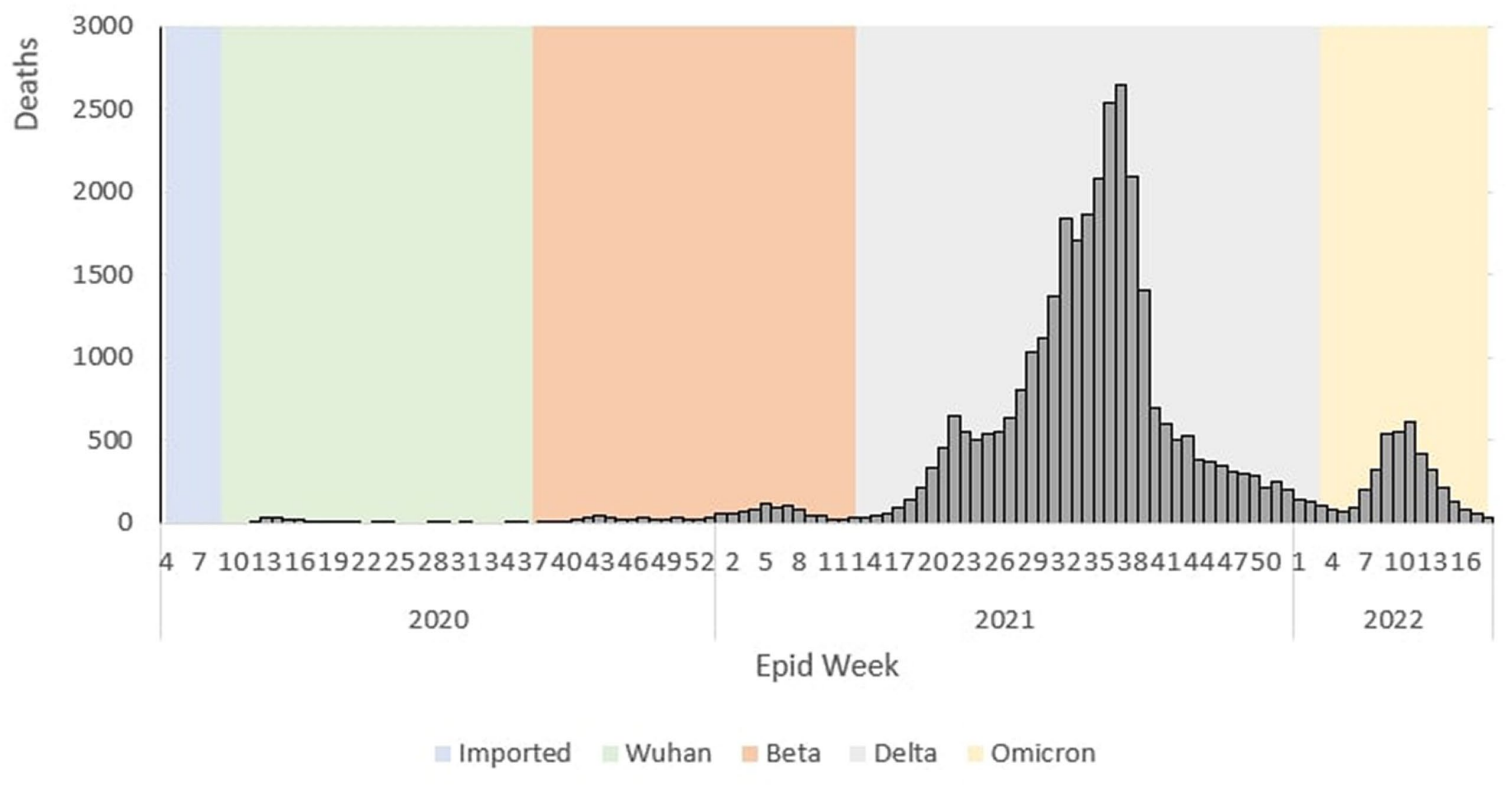
Weekly COVID-19 deaths by outbreaks in Malaysia, 2020 to 2022.

The epidemiological curve represents a sharp increase during the Wuhan outbreak, peaking at Ep Wk 14/2020 (*n* = 26). Following this, during the Beta outbreak, the epidemiological curve peaked at Ep Wk 5/2021 (*n* = 111). This was followed by the Delta outbreak, where the epidemiological curve peaked at Ep Wk 37/2021 (*n* = 2,647). Subsequently, during the Omicron outbreak, the epidemiological curve rose rapidly and peaked at Ep Wk 11/2022 (*n* = 609), as shown in Figure 4.

### Gender distribution of cases

#### Overall

Of the total 4,456,736 cases reported during the study period, 2,379,375 (53.39%) and 2,077,361 (46.61%) were males and females, respectively. The male-to-female cases ratio was 1.2. The COVID-19 gender-specific incidence rates were 141.87 and 130.78 per 1,000 males and females, respectively (Table 3).

**Table 3:** Gender distribution of COVID-19 cases and deaths by COVID-19 variants outbreaks in Malaysia.

		Wuhan			Beta			Delta			Omicron			Overall (Ep Wk 4/2020 to Ep Wk 18/2022)		
		Male	Female	Ratio	Male	Female	Ratio	Male	Female	Ratio	Male	Female	Ratio	Male	Female	Ratio
Cases	N	6,183	2,310	2.68	224,086	109,402	2.05	1,352,873	1,114,931	1.21	795,645	850,402	0.94	2,379,375	2,077,361	1.15
	%	72.80	27.20		67.19	32.81		54.82	45.18		48.34	51.66		53.39	46.61	
	IR	0.37	0.15		13.37	6.91		80.67	70.19		47.44	53.54		141.87	130.78	
Death	N	88	33	2.67	732	400	1.83	17,441	13,108	1.33	2,168	1,602	1.35	20,434	15,145	1.35
	%	72.73	27.27		64.66	35.34		57.09	42.91		57.51	42.49		57.43	42.57	
	DR	0.01	0.002		0.04	0.03		1.04	0.83		0.13	0.10		1.22	0.95	
	CFR	1.42	1.18		0.33	0.37		1.29	1.18		0.27	0.19		0.86	0.73	

#### Outbreaks

**Figure 5:**
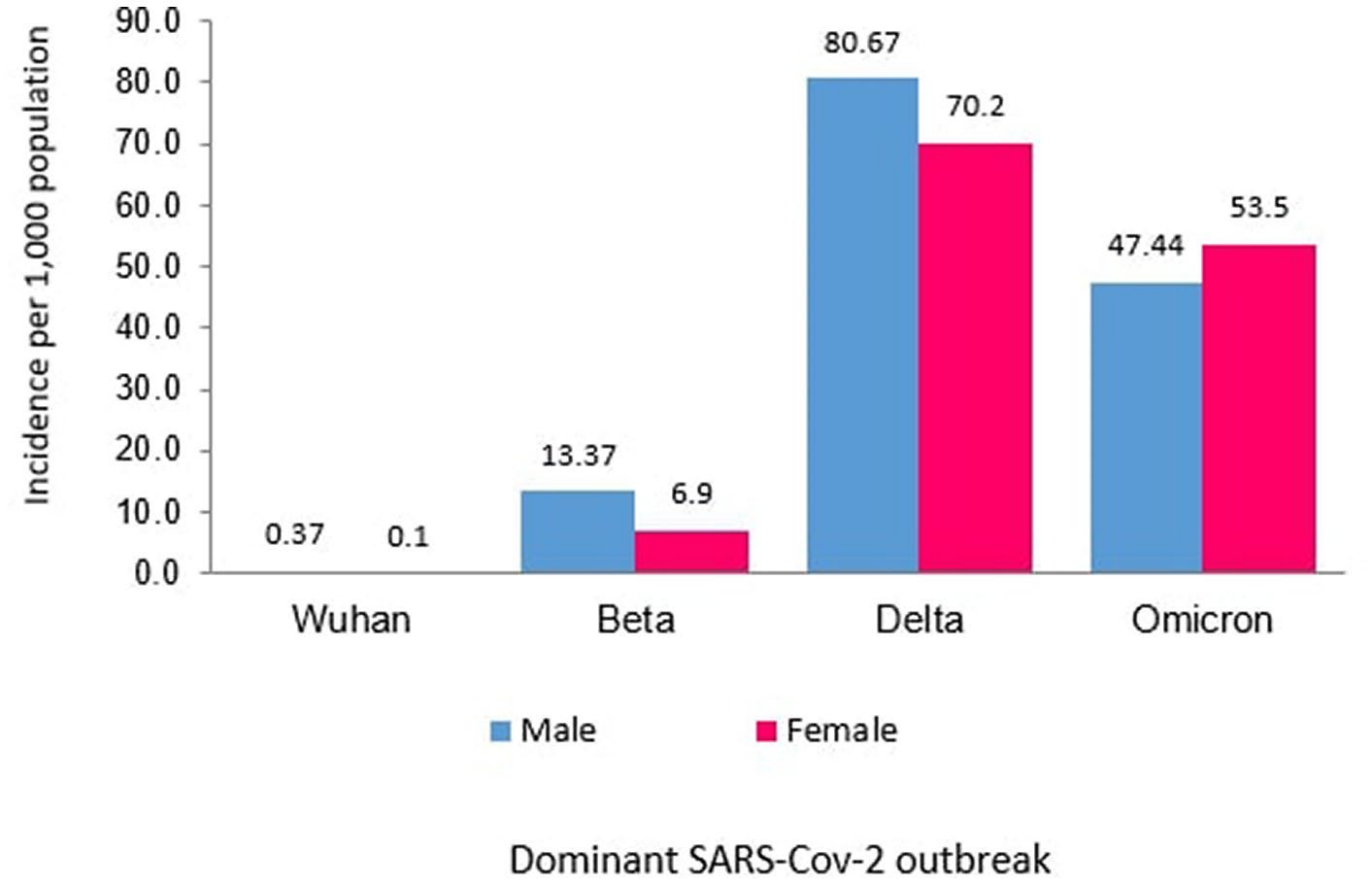
Male and female COVID-19 incidence rates (per 1,000 population) in Malaysia.

**Figure 6:**
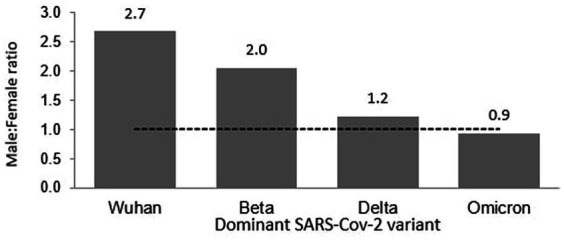
Male to female ratio of COVID-19 cases in Malaysia.

Of the 8,493 total cases reported during the Wuhan outbreak, 6,183 (72.80%) and 2,310 (27.20%) were males and females, respectively. The male-to-female cases ratio was 2.7. The COVID-19 gender-specific incidence rates per 1,000 populations for the Wuhan outbreak were 0.37 and 0.10 in males and females, respectively (Table 3; Figures 5, 6). During Beta outbreak, 333,488 cases were reported, with 224,086 (67.19%) and 109,402 (32.81%) males and females, respectively. The male-to-female cases ratio was 2.1. The COVID-19 gender-specific incidence rates per 1,000 populations for the Beta outbreak were 13.37 and 6.91 in males and females, respectively (Table 3; Figures 5, 6). Of the 2,467,804 total cases reported for the Delta outbreak, 1,352,873 (54.82%) and 1,114,931 (45.18%) were males and females, respectively. The male-to-female cases ratio was 1.2.

The COVID-19 gender-specific incidence rates per 1,000 populations for the Delta outbreak were 80.67 and 70.19 in males and females, respectively (Table 3; Figures 5, 6). As for the Omicron outbreak, 1,646,047 cases were reported, with 795,645 (48.34%) and 850,402 (51.66%) males and females, respectively. The ratio of male to female cases was 0.9. The COVID-19 gender-specific incidence rates per 1,000 populations for the Omicron outbreak were 47.44 and 53.5 in males and females, respectively (Table 3; Figures 5, 6). A higher number of COVID-19 cases and incidence rates were observed among males across all the outbreaks except for the Omicron outbreak, which reported higher numbers of COVID-19 cases and incidence rates among females (Figure 5). The male-to-female cases ratio reduced from the Wuhan outbreak to the Omicron outbreak (Figure 6).

### Gender distribution of deaths

#### Overall

Of the 35,579 total deaths reported during the study period, 20,434 (57.43%) and 15,145 (42.57%) were males and females, respectively. The male-to-female deaths ratio was 1.4. The COVID-19 gender-specific death rates per 1,000 populations during this study period were 1.22 and 0.95 in males and females, respectively. The COVID-19 gender-specific CFR rates during this study period were 0.86 and 0.73% in males and females, respectively (Table 3).

#### Outbreaks

Of the 121 total deaths reported during the Wuhan outbreak, 88 (72.73%) and 33 (27.27%) were males and females, respectively. The male-to-female deaths ratio was 2.7. The COVID-19 gender-specific death rates per 1,000 populations for the Wuhan outbreak were 0.01 and 0.002 in males and females, respectively. The COVID-19 gender-specific CFR rates for the Wuhan outbreak were 1.42 and 1.18% in males and females, respectively (Table 3). During the Beta outbreak, 1,132 total deaths were reported, with 732 (64.66%) and 400 (35.34%) being males and females, respectively. The ratio of male to female deaths was 1.8. The COVID-19 gender-specific death rates per 1,000 populations for the Beta outbreak were 0.04 and 0.03 in males and females, respectively. The COVID-19 gender-specific CFR rates for the Beta outbreak were 0.33 and 0.37% in males and females, respectively (Table 3). Of the 30,549 total deaths reported in the Delta outbreak, 17,441(57.09%) and 13,108 (42.91%) were males and females, respectively. The ratio of male to female deaths was 1.3.

**Figure 7:**
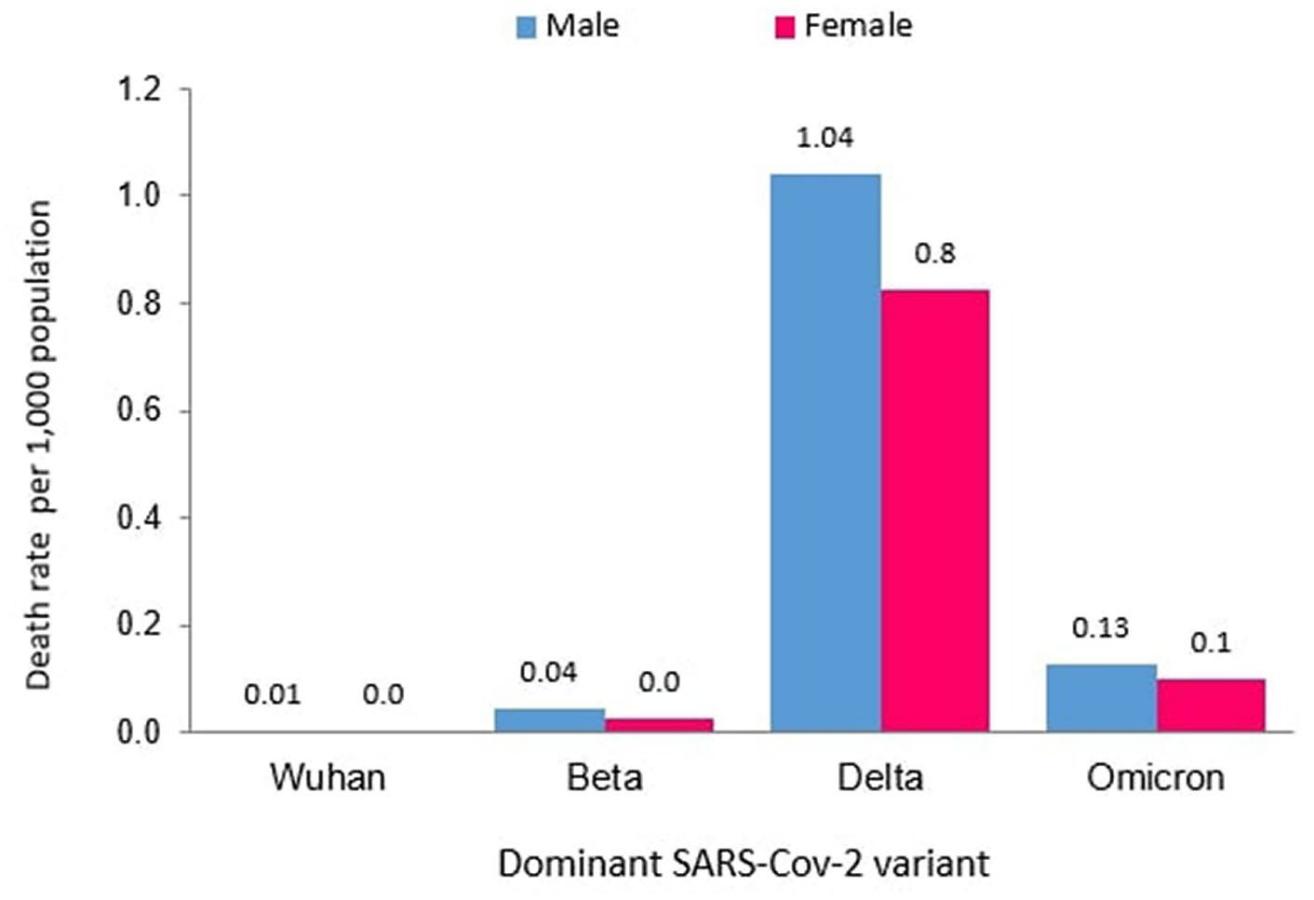
Male and female COVID-19 death rates (per 1,000 population) in Malaysia.

The COVID-19 gender-specific death rates per 1,000 populations for the Delta outbreak were 1.04 and 0.83 in males and females, respectively. The COVID-19 gender-specific CFR rates for the Delta outbreak were 1.29 and 1.18% in males and females, respectively (Table 3). As for the Omicron outbreak, 3,770 deaths were reported, with 2,168 (57.51%) and 1,602 (42.49%) males and females, respectively. The male-to-female deaths ratio was 1.4. The COVID-19 gender-specific death rates per 1,000 populations for the Omicron outbreak were 0.13 and 0.10 in males and females, respectively. The COVID-19 gender-specific CFR rates for the Omicron outbreak were 0.27 and 0.19% in males and females, respectively (Table 3; Figure 7). A higher number of COVID-19 deaths, death rate and CFR were observed among males across all the outbreaks except for the Beta outbreak, where a higher CFR was observed among females.

### Age distribution of cases

#### Overall

**Figure 8:**
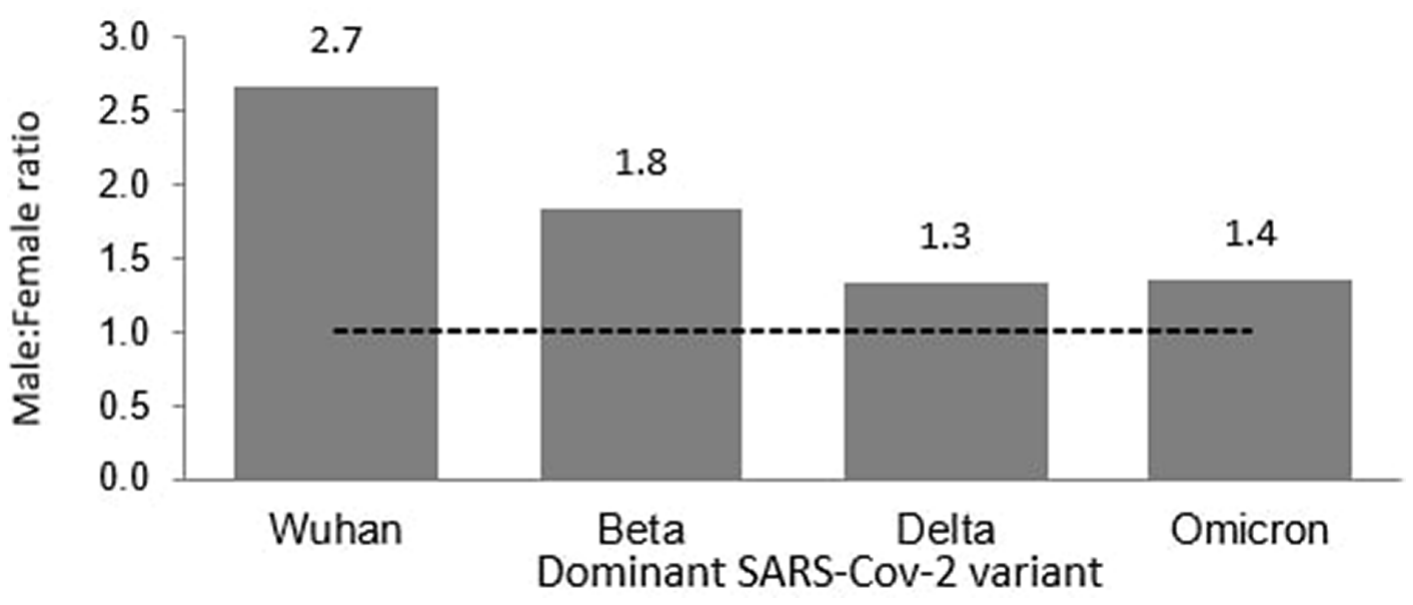
Male and female ratio of COVID-19 death Malaysia.

From the total of 4,456,736 cases reported during the study period, the age group with the highest and lowest cases were reported for individuals aged 20–29 years (*n* = 1,027,286) and 80+ years (*n* = 39,852), respectively. The proportion of COVID-19 cases by 10-year age during the study period are as follows: 0–9 years (11.26%), 10–19 years (11.73%), 20–29 years (23.50%), 30–39 years (22.19%), 40–49 years (13.20%), 50–59 years (8.98%), 60–69 years (5.78%), 70–79 years (2.46%) and more than 80 years (0.91%) as shown in Table 4. The COVID-19 age-specific incidence rates per 1,000 populations by the 10-year age groups for the study period were highest and lowest for individuals ages 30–39 years (incidence rates 177.21) and 10–19 years (incidence rates 97.7), respectively (Figure 8).

**Table 4:** Age distribution of COVID-19 cases and deaths by COVID-19 variants outbreaks in Malaysia.

Age group	Wuhan					Beta					Delta					Omicron					Overall (Ep Wk 4/2020 to Ep Wk 18/2022)				
	Cases	*IR	Death	**DR	CFR	Cases	*IR	Death	**DR	CFR	Cases	*IR	Death	**DR	CFR	Cases	*IR	Death	**DR	CFR	Cases	*IR	Death	**DR	CFR
0-9	259 (3.35)	0.05	0 (0.00)	0.00	-	19,613 (6.23)	3.86	7 (0.62)	0.00	0.0	292,456 (12.12)	58.08	64 (0.21)	0.01	0.0	179,844 (10.99)	35.71	36 (0.95)	0.01	0.0	492,225 (11.26)	97.75	107 (0.30)	0.02	0.0
10–19	844 (10.93)	0.16	0 (0.00)	0.00	-	24,687 (7.84)	4.63	5 (0.44)	0.00	0.0	300,988 (12.48)	57.34	75 (0.25)	0.01	0.0	186,291 (11.39)	35.49	26 (0.69)	0.00	0.0	512,881 (11.73)	97.70	106 (0.30)	0.02	0.0
20–29	2,138 (27.69)	0.34	3 (2.48)	0.00	0.1	96,346 (30.59)	15.16	17 (1.50)	0.00	0.0	548,620 (22.74)	87.31	686 (2.25)	0.11	0.1	379,988 (23.23)	60.47	47 (1.25)	0.01	0.0	1,027,286 (23.50)	163.48	753 (2.12)	0.12	0.1
30–39	1,523 (19.73)	0.28	9 (7.44)	0.00	0.6	86,013 (27.31)	15.84	40 (3.53)	0.01	0.0	503,471 (20.87)	91.97	2,260 (7.40)	0.41	0.4	378,908 (23.16)	69.21	129 (3.42)	0.02	0.0	970,141 (22.19)	177.21	2,438 (6.85)	0.45	0.3
40–49	943 (12.21)	0.25	9 (7.44)	0.00	1.0	42,833 (13.60)	11.32	93 (8.22)	0.02	0.2	312,325 (12.95)	81.29	4,244 (13.89)	1.10	1.4	220,714 (13.49)	57.45	269 (7.14)	0.07	0.1	576,963 (13.20)	150.18	4,615 (12.97)	1.20	0.8
50–59	1,004 (13.00)	0.32	19 (15.70)	0.01	1.9	25,406 (8.07)	8.20	191 (16.87)	0.06	0.8	223,100 (9.25)	71.47	5,884 (19.26)	1.88	2.6	143,069 (8.74)	45.83	427 (11.33)	0.14	0.3	392,673 (8.98)	125.79	6,521 (18.33)	2.09	1.7
60–69	715 (9.26)	0.34	38 (31.40)	0.02	5.3	13,585 (4.31)	6.44	327 (28.89)	0.16	2.4	147,598 (6.12)	67.69	7,153 (23.41)	3.28	4.8	90,698 (5.54)	41.60	737 (19.55)	0.34	0.8	252,654 (5.78)	115.88	8,258 (23.21)	3.79	3.3
70–79	211 (2.73)	0.21	25 (20.66)	0.02	11.8	4,815 (1.53)	4.75	256 (22.61)	0.25	5.3	61,818 (2.56)	57.74	5,842 (19.12)	5.46	9.5	40,528 (2.48)	37.85	952 (25.25)	0.89	2.3	107,401 (2.46)	100.31	7,079 (19.90)	6.61	6.6
80+	84 (1.09)	0.22	18 (14.88)	0.05	21.4	1,708 (0.54)	4.51	196 (17.31)	0.52	11.5	22,051 (0.91)	55.54	4,341 (14.21)	10.93	19.7	15,994 (0.98)	40.29	1,147 (30.42)	2.89	7.2	39,852 (0.91)	100.38	5,702 (16.03)	14.36	14.3
Total	7,721	2.17	121	0.10		315,006	74.71	1,132	1.02		2,412,427	628.42	30,549	23.21		1,636,034	423.90	3,770	4.37		4,372,076	1,128.67	35,579	28.66	

IR = age-specific incidence rate per 1,000 populations. DR = age-specific deaths per 1,000 populations. Denominators for cases and deaths population estimates sourced from Department of Statistics Malaysia website (http://pqi.stats.gov.my). *Incidence of COVID-19 in 2020–2021 per 1,000 population [(Total 2020 and 2021 cases)/(Total populations of 2020 and 2021)*1,000]. **DR = Deaths of COVID-19 in 2020–2021 per 1,000 population. Values for cases and death are number and %. Values for CFR are %. Missing age data for cases and deaths in Wuhan strain, *n* = 772. Missing age data for cases and deaths in Beta strain, *n* = 18,482. Missing age data for cases and deaths in Delta strain, *n* = 55,377. Missing age data for cases and deaths in Omicron strain, *n* = 10,013. Missing age data for cases and deaths in Overall cases, *n* = 84,660.

#### Outbreaks

**Figure 9:**
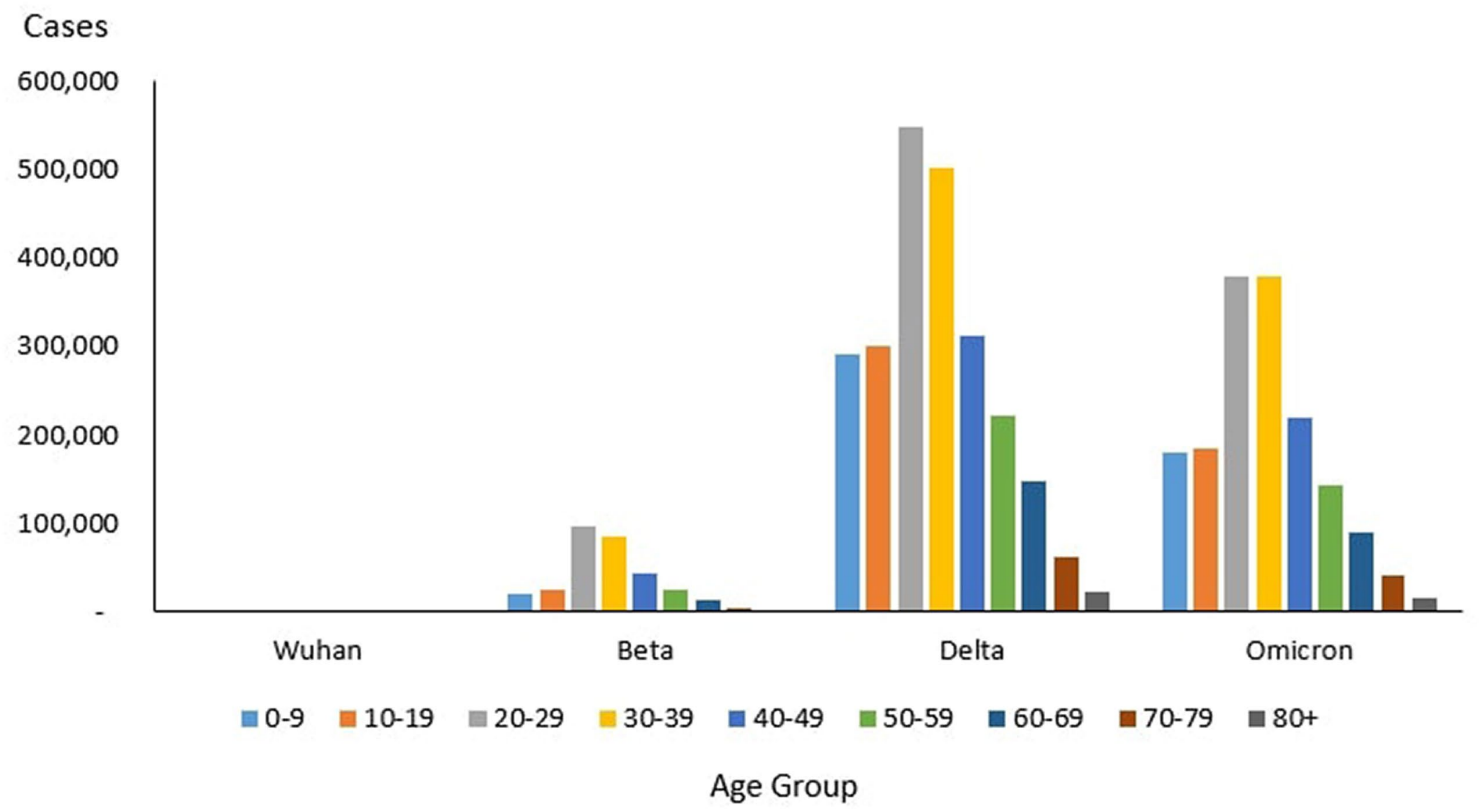
Number of COVID-19 cases by age group, Malaysia 2020-2022.

**Figure 10:**
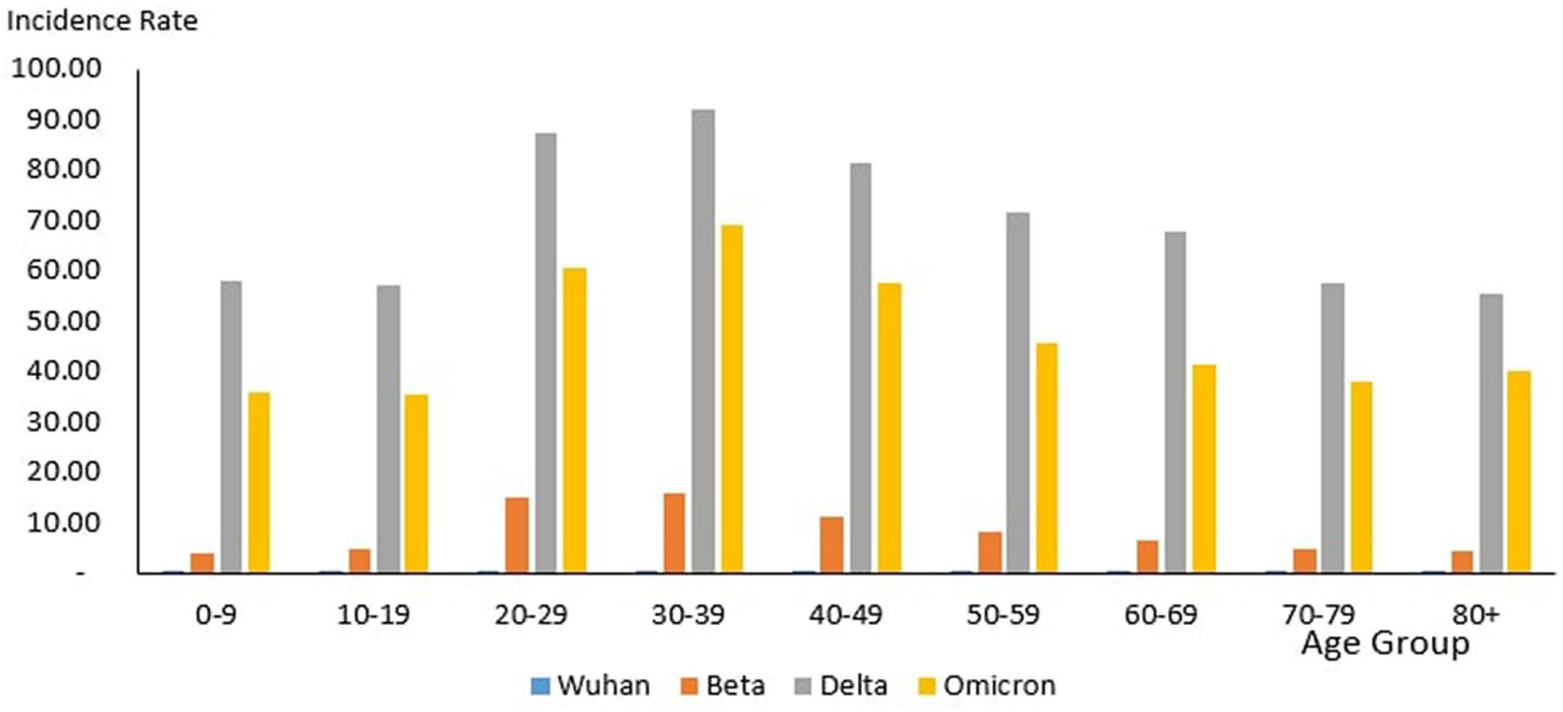
Age-specific COVID-19 incidence rate (per 1,000 population), Malaysia 2020-2022.

Of the 8,493 cases reported during the Wuhan outbreak, the highest and lowest cases were in the age group 20–29 years (*n* = 2,138) and 80+ years (*n* = 84), respectively. The proportion of COVID-19 cases by 10-year age group for the Wuhan outbreak are as follows: 0–9 years (3.35%), 10–19 years (10.93%), 20–29 years (27.69%), 30–39 years (19.73%), 40–49 years (12.21%), 50–59 years (13.0%), 60–69 years (9.26%), 70–79 years (2.73%), and 80+ years (1.09%). The COVID-19 age-specific incidence rates per 1,000 populations in 10-year age groups during the Wuhan outbreak were highest for individuals ages 20–29 years and 60–69 years (incidence rates 0.34) and lowest for individuals ages 0–9 years (incidence rates 0.05) respectively (Table 4; Figures 9, 10).

During the Beta outbreak, 333,488 cases were reported, with the highest and lowest cases in the age group 20–29 years (*n* = 96,346) and 80+ years (*n* = 1,708), respectively. The proportion of COVID-19 cases by 10-year age group for the Beta outbreak are as follows: 0–9 years (6.23%), 10–19 years (7.84%), 20–29 years (30.59%), 30–39 years (27.31%), 40–49 years (13.6%), 50–59 years (8.07%), 60–69 years (4.31%), 70–79 years (1.53%) and 80+ years (0.54%). The COVID-19 age-specific incidence rates per 1,000 populations in 10-year age groups during the Beta outbreak were highest for individuals ages 30–39 years (incidence rates 15.84) and lowest for individuals ages 0–9 years (incidence rates 3.86) respectively (Table 4; Figures 9, 10).

During the Delta outbreak, 2,467,804 cases were reported, with the highest and lowest cases in the age group 20–29 years (*n* = 548,620) and 80+ years (*n* = 2,251), respectively. The proportion of COVID-19 cases by 10-year age group for the Delta outbreak are as follows: 0–9 years (12.12%), 10–19 years (12.48%), 20–29 years (22.74%), 30–39 years (20.87%), 40–49 years (12.95%), 50–59 years (9.25%), 60–69 years (6.12%), 70–79 years (2.56%) and 80+ years (0.91%). The COVID-19 age-specific incidence rates per 1,000 populations in 10-year age groups during the Delta outbreak were highest for individuals ages 30–39 years (incidence rates 91.97) and lowest for individuals ages 80+ years (incidence rates 55.54) respectively (Table 4; Figures 9, 10).

**Figure 11:**
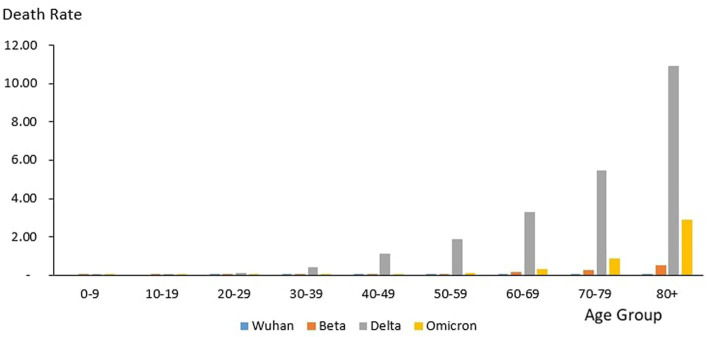
Age-specific COVID-19 death rate (per 1,000 population), Malaysia 2020-2022.

**Figure 12:**
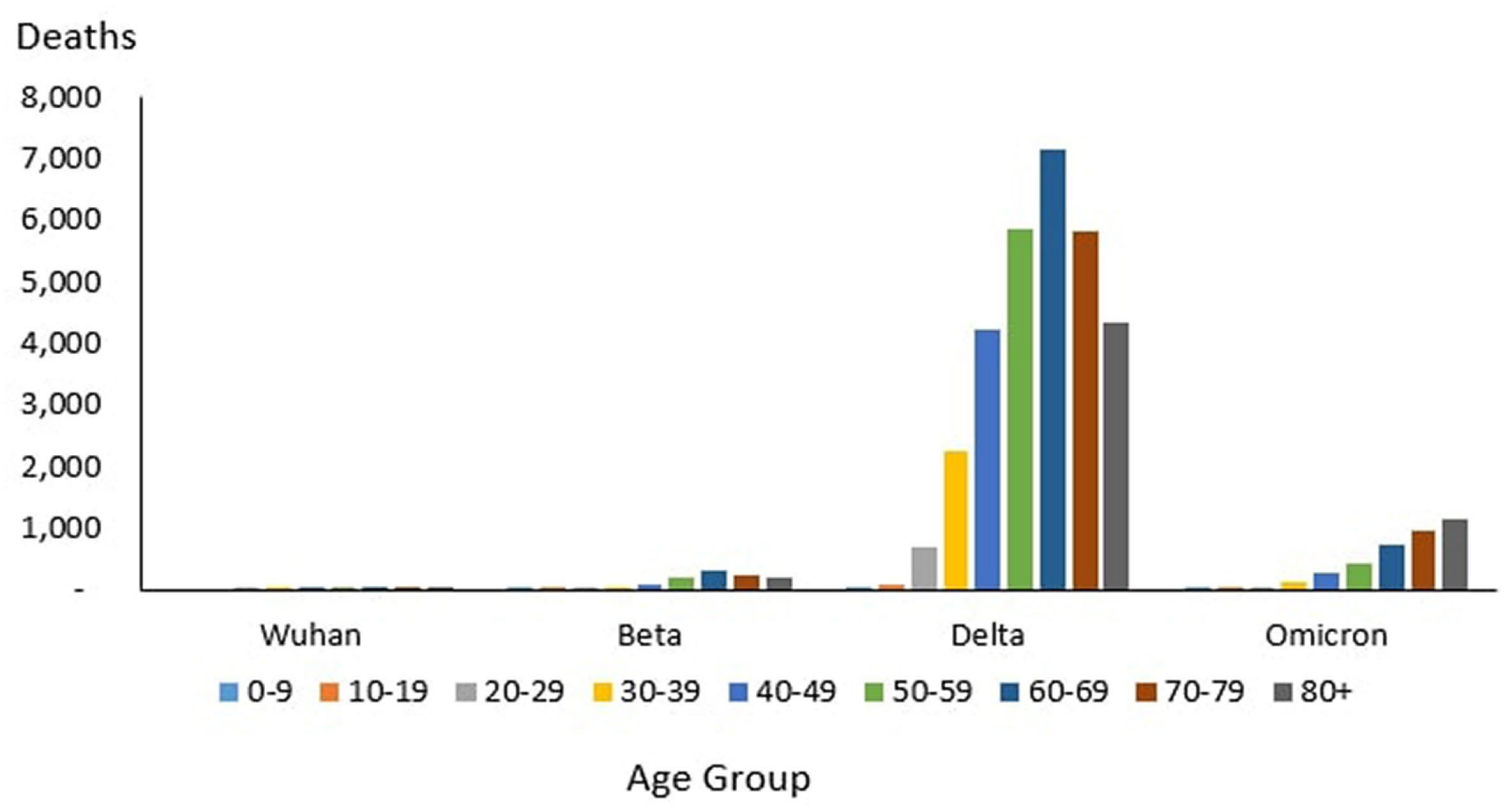
Age-specific COVID-19 death, Malaysia 2020-2022.

Of the 1,646,047 cases reported during the Omicron outbreak, the age group with the highest and lowest cases were 20–29 years (*n* = 379,988) and 80+ years (*n* = 15,994), respectively. The proportion of COVID-19 cases by 10-year age group for the Omicron outbreak are as follows: 0–9 years (10.99%), 10–19 years (11.39%), 20–29 years (23.23%), 30–39 years (23.16%), 40–49 years (13.49%), 50–59 years (8.74%), 60–69 years (5.54%), 70–79 years (2.48%) and 80+ years (0.98%). The COVID-19 age-specific incidence rates per 1,000 populations in 10-year age groups during the Omicron outbreak were highest for individuals ages 30–39 years (incidence rates 69.21) and lowest for individuals ages 10–19 years (incidence rates 35.49), respectively (Table 4; Figures 11, 12).

Across all the outbreaks, the highest and lowest number of COVID-19 cases by age was reported for individuals aged 20–29 and over 80 years, respectively. The number of COVID-19 cases by age starts to reduce among individuals ages 30 and above across all the outbreaks (Figure 8). For all the outbreaks, the highest COVID-19 age-specific incidence rate was reported among individuals aged 30–39 years, except for the Wuhan outbreak, which had the highest incidence rate among individuals aged 20–29 years and 60–69 years, respectively. The lowest COVID-19 age-specific incidence rate was reported among individuals in the age groups 0–9 years and 10–19 years for all outbreaks except for the Delta outbreak, which reported the lowest COVID-19 age-specific incidence rate among individuals in the age group more than 80 years (Figure 10).

### Age distribution of death

#### Overall

From the total of 35,579 deaths reported during the study period, the highest and lowest deaths were reported in the age group 60–69 years (*n* = 8,258) and 10–19 years (*n* = 106), respectively. The proportion of COVID-19 deaths by 10-year age group during the study period is as follows: 0–9 years (0.3%), 10–19 years (0.3%), 20–29 years (2.12%), 30–39 years (6.85%), 40–49 years (12.97%), 50–59 years (18.33%), 60–69 years (23.21%), 70–79 years (19.90%) and 80+ years (16.03%). The COVID-19 age-specific death rates per 1,000 populations in 10-year age groups during the study period were highest for individuals ages 80+ years (death rates 14.36) and lowest for individuals ages 0–9 years and 10–19 years (death rates 0.02), respectively. The COVID-19 age-specific CFR rates in 10-year age groups during the study period were highest for individuals ages 80+ years (CFR 14.3%) and lowest for individuals ages 0–9 years and 10–19 years (CFR 0%) respectively (Table 4).

#### Outbreaks

From the total of 121 deaths reported during the Wuhan outbreak, the highest and lowest deaths were reported in the age group 60–69 years (*n* = 38) and the group 0–9 years and 10–19 years (*n* = 0), respectively. The proportion of COVID-19 deaths by 10-year age group for the Wuhan outbreak is as follows: 0–9 years (0.0%), 10–19 years (0.0%), 20–29 years (2.48%), 30–39 years (7.44%), 40–49 years (7.44%), 50–59 years (15.7%), 60–69 years (31.4%), 70–79 years (20.66%) and 80+ years (14.88%). The COVID-19 age-specific death rates per 1,000 populations in 10-year age groups for the Wuhan outbreak were highest for individuals ages 80+ years (death rates 0.05) and lowest for the age group between 20 and 29 years (death rates 0.0005), respectively. The COVID-19 age-specific CFR rates in 10-year age groups for the Wuhan outbreak were highest for individuals ages 80+ years (CFR 21.4%) and lowest for individuals ages 20–29 years (CFR 0.1%), respectively (Table 4; Figure 11).

During the Beta outbreak, a total of 1,132 deaths were reported, with the highest and lowest deaths noted in the age group 60–69 years (*n* = 327) and 10–19 years (*n* = 5), respectively. The proportion of COVID-19 deaths by 10-year age group for the Beta outbreak are as follows: 0–9 years (0.62%), 10–19 years (0.44%), 20–29 years (1.50%), 30–39 years (3.53%), 40–49 years (8.22%), 50–59 years (16.87%), 60–69 years (28.89%), 70–79 years (22.61%) and 80+ years (17.31%). The COVID-19 age-specific death rates per 1,000 populations in 10-year age groups for the Beta outbreak were highest and lowest for individuals ages 80+ years (death rates 0.52) and 0–9 years and 10–19 years (death rates 0.001), respectively. The COVID-19 age-specific CFR rates in 10-year age groups for Beta outbreak were highest for individuals ages 80+ years (CFR 11.5%) and lowest for individuals ages 0–39 years (CFR 0.1%), respectively (Table 4; Figure 11).

During the Delta outbreak, 30,549 deaths were reported, with the highest and lowest deaths in the age group of 60–69 years (*n* = 7,153) and 0–9 years (*n* = 64), respectively. The proportion of COVID-19 deaths by 10-year age group for the Delta outbreak are as follows: 0–9 years (0.21%), 10–19 years (0.25%), 20–29 years (2.25%), 30–39 years (7.40%), 40–49 years (13.89%), 50–59 years (19.26%), 60–69 years (23.41%), 70–79 years (19.12%) and 80+ years (14.21%). The COVID-19 age-specific death rates per 1,000 populations in 10-year age groups for the Delta outbreak were highest for individuals ages 80 + years (death rates 10.93) and lowest for individuals aged 0–9 years and 10–19 years (death rates 0.01), respectively. The COVID-19 age-specific CFR rates in 10-year age groups for the Delta outbreak were highest for individuals ages 80+ years (CFR 19.7%) and lowest for individuals ages 0–9 and 10–19 years (CFR 0.0%) respectively (Table 4; Figure 11).

Of the 3,770 deaths reported during the Omicron outbreak, the age group with the highest number of deaths was 80+ years (*n* = 1,147), and the lowest was 10–19 years (*n* = 26), respectively. The proportion of COVID-19 deaths by 10-year age group for the Omicron outbreak are as follows: 0–9 years (0.95%), 10–19 years (0.69%), 20–29 years (1.25%), 30–39 years (3.42%), 40–49 years (7.14%), 50–59 years (11.33%), 60–69 years (19.55%), 70–79 years (25.25%) and 80+ years (30.42%). The COVID-19 age-specific death rates per 1,000 populations in 10-year age groups for the Omicron outbreak were highest for individuals aged 80+ years (death rates 2.89) and lowest for individuals aged 10–19 years (death rates 0.0), respectively. The COVID-19 age-specific CFR rates in 10-year age groups for Omicron outbreak were highest for individuals ages 80+ years (CFR 7.2%) and lowest for individuals ages 0–39 years (CFR 0.0%), respectively (Table 4; Figure 11).

Across all the outbreaks, the highest and lowest number of COVID-19 deaths by age was reported among individuals in the age groups of 60–69 years and 0–19 years, respectively, except for the Omicron outbreak, which reported the highest number of COVID-19 death by age among individual age group 80 and above. The number of COVID-19 deaths by age starts to reduce among individuals ages 70 and above across all the outbreaks (Figure 9) except during the Omicron outbreak, in which COVID-19 deaths tend to increase with advancing age. Across all the outbreaks, the highest COVID-19 age-specific death rate was reported among individuals in the age groups of 80 and above years for all the outbreaks. The lowest COVID-19 age-specific death rate was reported among individuals in the age groups 10–19 years for all outbreaks except for the Wuhan outbreak, which reported the lowest COVID-19 age-specific death rate among individuals in the age group of 20–29 years (Figure 9). Similarly, the highest age-specific CFR was reported among individuals aged 80 years and above across all the outbreaks.

### Testing and test positivity rate

#### Overall

A total of 58,906,954 COVID-19 tests were conducted during the study period in which the highest weekly COVID-19 total test (RT-PCR and RTK-AG) and the total test positivity rate (RT PCR and RTK Ag) was reported at 1,788,410 tests (Ep Wk7/2022) and 7.57%, respectively, (Table 2).

#### Outbreaks

During the Wuhan outbreak, a total of 853,379 (1.5%) tests were done, wherein the highest (*n* = 86,018) and lowest (*n* = 586) weekly COVID-19 total test was reported in Ep Wk18/2020 and Ep Wk 9, respectively. The test positivity rate was reported at 1.0% during this period. As for the Beta outbreak, a total of 8,781,144 (14.91%) tests were done where the highest (*n* = 629,225) and lowest (*n* = 51,673) weekly COVID-19 total test were reported in Ep Wk 3/2021 and Ep Wk 37/2020, respectively. The test positivity rate was reported at 3.8% during this period.

In the time of the Delta outbreak, a total of 33,603,291(57.04%) tests were done where the highest (*n* = 1,119,105) and lowest (*n* = 231,648) weekly COVID-19 total tests were reported in Ep Wk 33/2021 and Ep Wk 3/2022, respectively. The test positivity rate was reported at 7.34% during this period. During the Omicron outbreak, a total of 15,157,523 (25.73%) tests were done where the highest (*n* = 1,788,410) and lowest (*n* = 221,636) weekly COVID-19 total tests were reported in Ep Wk 7/2022 and Ep Wk 18/2022, respectively. The test positivity rate was reported at 10.86%, as shown in Table 2. The Wuhan, Beta, Delta, and Omicron outbreak test positivity rates were 0.995, 3.798, 7.344, and 10.860, respectively.

#### Trends

**Figure 13:**
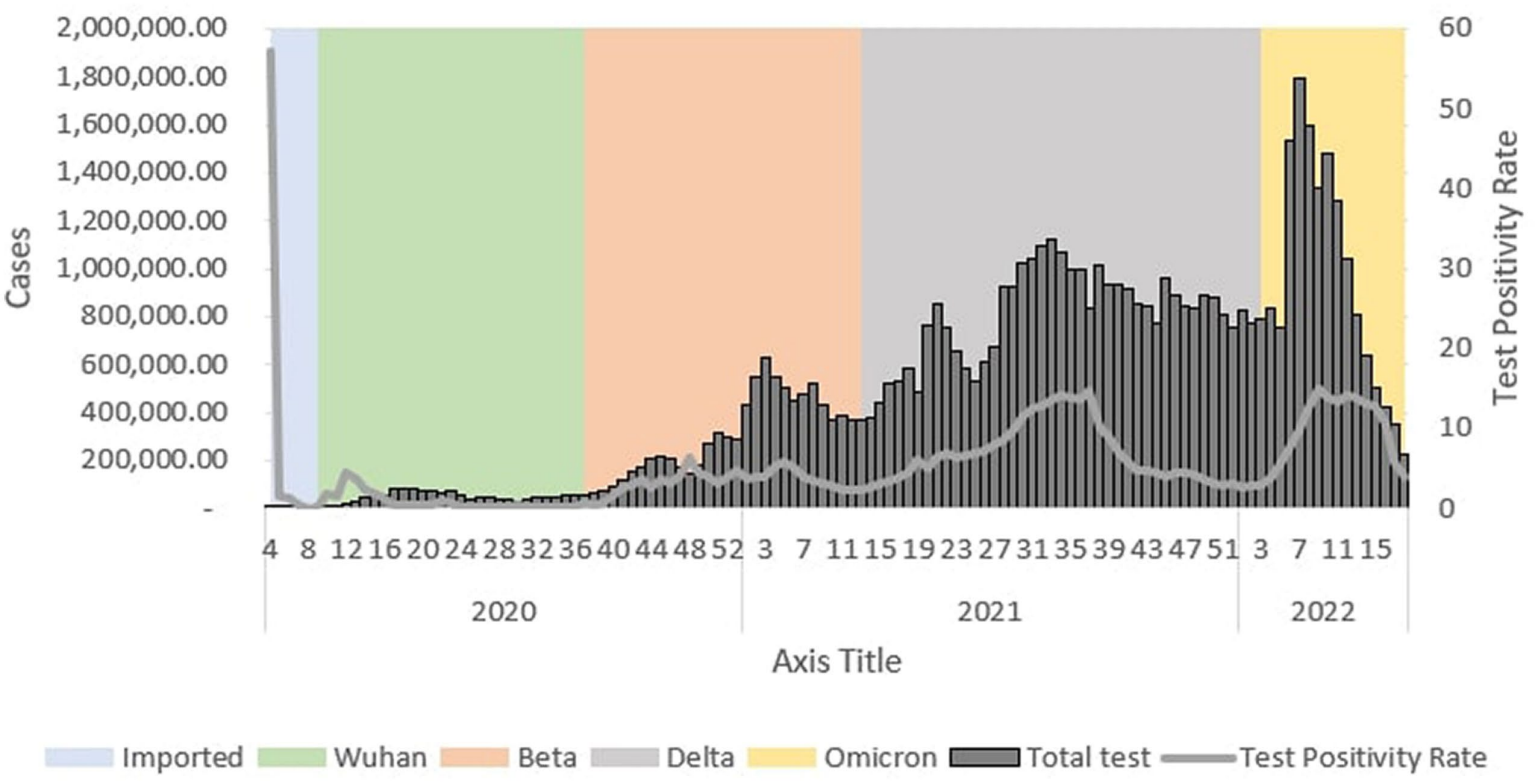
Weekly total COVID-19 tests and test positivity rate by outbreaks in Malaysia, 2020-2022.

During the Wuhan outbreak, the number of tests increased from Ep Wk 10 (*n* = 3,904) and peaked in Ep Wk 18 (*n* = 86,018). Subsequently, the number of tests decreased from Ep Wk 19 (*n* = 82,072) to Ep Wk 25 (*n* = 40,609). Following this, the Beta outbreak began, where testing trends started to increase from Ep Wk 38/2020 (*n* = 66,922) and peaked in Ep Wk 3/2021 (*n* = 629,225). Subsequently, a downward testing trend has been observed from Ep Wk 4/2021 (*n* = 544,745) till Ep Wk 13/2021 (*n* = 369,701). This was followed by the Delta outbreak, wherein the testing trends started to increase from Ep Wk 14/2021 (*n* = 382,171) and peaked in Ep Wk 33/2021 (*n* = 1,119,015), which corresponded to the longest increase of testing trends of 33 weeks. A downward trend of testing has been observed from Ep Wk 34/2021 (*n* = 1,071,056) till Ep Wk 3/2022 (*n* = 776,217), corresponding to the longest decrease in the testing trend of 23 weeks. Following this, the Omicron outbreak began where a testing trend started to increase from Ep Wk 4/2022 (*n* = 831,806) and peaked at Ep Wk 7/2022 (*n* = 1,788,410), which was followed by a downward trend of testing from Ep Wk 8/2022 (*n* = 1,596,093) to Ep Wk 18/2022 (*n* = 221,636) as shown in Figure 13.

## Discussion

This study described the COVID-19 case demographics, hospital admissions and testing capacities for the four outbreaks, namely the Wuhan, Beta, Delta and Omicron, from the beginning of the outbreak in January 2020 till it was declared endemic in April 2022. A total of 4,456,736 cases was analyzed in this study, which showed that the Delta and Omicron outbreaks reported the highest numbers of COVID-19 cases, which was also found in studies in India and Bangladesh (21–25). These findings can be explained by the increased transmissibility in the Delta and Omicron variants of the COVID-19 virus. Moreover, studies have reported that the Delta variant is 50% more transmissible than the Alpha, Beta or Wuhan variants (26). In addition, a higher number of infections that occurred during the Delta and Omicron outbreaks can be attributed to the longer outbreak duration of these variants. Furthermore, the easing of movement control measures resulted in increased travel and population mobility during the Delta and Omicron outbreaks, which allowed for further spread of the disease. Finally, increased testing capacity during the Delta and Omicron outbreaks led to higher case detection, resulting in increased case numbers. This higher transmissibility, longer outbreak period and increased population mobility and testing contributed to conditions that caused the massive spread of the disease, causing extreme suffering of the populations as well as overwhelming the healthcare systems (27). The presence of highly transmissible variants would require an urgent need for the health systems to closely monitor the outbreak and promptly institute outbreak control measures (28).

This study also found higher COVID-19 incidence among males aged between 20 and 39. This finding can be explained by individuals in this age group being more socially active, therefore having an increased risk of being infected. In addition, differences in behavior and immunological factors may also explain the increased prevalence of the disease in this population (29). This could be attributed to high-risk behaviors such as smoking and alcohol consumption among males, resulting in more severe forms of COVID-19 disease (30). Studies conducted by the Center for Disease Control (CDC) in the United States also showed similar findings affecting males in this age group to have higher infection rates and deaths (30–32).

This study showed that the highest number of patients requiring ICU admission, ventilator support, and deaths occurred during the Delta outbreak as compared to the other outbreaks. The increased morbidity and mortality of the Delta variant can be attributed to higher virulence, resulting in more severe forms of the disease (33). In addition, the longer duration of the Delta outbreak, causing a prolonged, sustained combination of high case numbers with more severe forms of the disease, would overwhelm the healthcare system, limiting the availability of the healthcare system to provide patient care, which would ultimately increase case mortality (33–35). The increased morbidity and mortality of the Delta variant were also observed in a meta-analysis involving 16 countries worldwide, including studies in the US, UK and India, where this finding was attributed to the variant’s higher disease severity and transmissibility (36–38).

The CFR during the Delta outbreak was reported at 1.24, which corresponded to the second-highest CFR during the study period. While the highest CFR was reported during the Wuhan outbreak at 1.42, the high CFR during the Wuhan outbreak is an effect of limited testing with a low detection rate, which biased the high observed CFR during this period. Interestingly, a reduction in trends of COVID-19 patients requiring ICU admission, ventilator support and mortality was observed as the Delta outbreak progressed. This can be attributed to the vaccination programs that had resulted in 70% of the population completing two doses of COVID-19 vaccination as of mid-2022 in Malaysia (39, 40).

A cumulative of 58,906,954 COVID-19 tests were conducted during the pandemic period, and it was observed that the test positivity rate has been increasing since the beginning of the outbreak in Malaysia. The test positivity rate for the different outbreaks was reported for the Wuhan (1.0), Beta (3.8), Delta (7.34), and Omicron (10.86) outbreaks, respectively. In addition, this study found that the highest number of COVID-19 tests was reported during the Delta outbreak in Malaysia. The reason could be due to the longer duration of the Delta outbreak (*n* = 43 weeks) compared to other outbreaks and the increased transmissibility of the virus. The longer outbreak duration allows more testing to be conducted due to increased testing capacity during the Delta outbreak (41–44) Furthermore, there was an increased requirement for COVID-19 testing for certain socio-economic activities such as return to work, travel and pre-operative procedures during the Delta outbreak and with the introduction of simple self-testing via the rapid antigen test made accessible to the public (45–47). The weekly COVID-19 test requirement since August 2021 by the health authorities for back-to-work employees to prevent the spread of COVID-19 has also greatly contributed to the increase in testing done nationally (45–47).

## Conclusion

The Delta outbreak was the most severe compared to other outbreaks during the study period in Malaysia. This is evident by the highest number of COVID-19 cases, ICU admissions, ventilatory requirements and deaths observed during the Delta outbreak. In addition, this study provides evidence that outbreaks of COVID-19, which are caused by highly virulent and transmissible variants, tend to be more severe and devastating if they are not controlled early on. This was the first attempt to describe the COVID-19 outbreak in detail. Hence, close monitoring of key epidemiological indicators, as reported in this study, is essential in the control and management of future COVID-19 outbreaks in Malaysia.

## Data availability statement

The raw data that supports the findings of this study are available on request from the corresponding author.

## Ethics statement

The study was registered with the National Medical Research Register (NMRR ID-22-00940-PF6).

## Author contributions

MM: Writing – review & editing, Writing – original draft, Visualization, Software, Resources, Project administration, Methodology, Investigation, Funding acquisition, Data curation, Conceptualization. AM: Writing – review & editing, Writing – original draft, Methodology, Investigation, Formal analysis, Data curation, Conceptualization. SS: Writing – review & editing, Writing – original draft, Supervision, Project administration, Methodology, Investigation, Data curation, Conceptualization. MS: Writing – review & editing, Project administration, Methodology, Investigation, Formal analysis, Conceptualization. CL: Writing – review & editing, Visualization, Resources, Project administration, Methodology, Investigation, Formal analysis, Data curation. CT: Writing – review & editing, Visualization, Methodology, Investigation, Formal analysis, Data curation. TA: Writing – review & editing, Visualization, Supervision, Project administration. HM: Writing – review & editing, Validation, Supervision, Methodology, Investigation. BS: Writing – review & editing, Writing – original draft, Visualization, Validation, Supervision, Methodology. N’AM: Writing – review & editing, Writing – original draft, Methodology, Investigation, Data curation, Conceptualization. NM: Writing – review & editing, Writing – original draft, Visualization, Software, Resources, Methodology, Investigation. ML: Writing – review & editing, Methodology, Investigation. LA: Writing – review & editing, Methodology, Investigation. MK: Writing – review & editing, Visualization, Methodology, Investigation, Formal analysis. NA: Writing – review & editing, Methodology, Investigation, Data curation. KT: Writing – review & editing, Visualization, Validation, Supervision, Methodology, Investigation. AZ: Writing – review & editing, Writing – original draft, Software, Project administration, Methodology, Investigation.

## Footnotes

^1^ https://github.com/MoH-Malaysia/covid19-public

^2^ https://covid-19.moh.gov.my/
